# In Vitro Fermentation Characteristics of Dietary Fibers Using Fecal Inoculum from Dogs Consuming a Dried Brewers Yeast Product

**DOI:** 10.3390/ani15213117

**Published:** 2025-10-27

**Authors:** Vanessa M. De La Guardia Hidrogo, Patricia M. Oba, Dalton A. Holt, Laura L. Bauer, Lindsey M. Rummell, Ryan N. Dilger, Kelly S. Swanson

**Affiliations:** 1 Department of Animal Sciences, University of Illinois Urbana-Champaign, Urbana, IL 61801, USA; vmd2@illinois.edu (V.M.D.L.G.H.); obapm@illinois.edu (P.M.O.); daholt2@illinois.edu (D.A.H.); llbauer@illinois.edu (L.L.B.); rdilger2@illinois.edu (R.N.D.); 2 Wilbur-Ellis Nutrition, Buhl, ID 83316, USA; lrummell@uoguelph.ca; 3 Division of Nutritional Sciences, University of Illinois Urbana-Champaign, Urbana, IL 61801, USA; 4 Department of Veterinary Clinical Medicine, University of Illinois Urbana-Champaign, Urbana, IL 61801, USA

**Keywords:** canine gastrointestinal health, canine microbiota, fecal metabolites

## Abstract

The increasing demand for functional ingredients has drawn attention to yeast and yeast-derived ingredients in pet foods. This experiment aimed to evaluate the in vitro fermentation characteristics of common dietary fibers using fecal inoculum from dogs fed either a control diet (CTRL) or a diet supplemented with dried brewers yeast at 1.5% inclusion (BY). Sixteen healthy adult dogs were acclimated for 7 d and then randomly assigned to the two treatments (*n* = 8/group) for 21 d. Fecal samples collected at the end of the treatment period, were preserved in 20% glycerol and later used as inoculum for in vitro fermentation of beet pulp, pectin, and cellulose over 0, 6, 12, or 18 h. Fermentation patterns differed among fiber substrates. Pectin was highly fermentable, beet pulp showed moderate fermentation, and cellulose was minimally fermented, as indicated by SCFA production and pH changes. Within each fiber, BY inocula produced more SCFA and a lower pH than CTRL after 18 h, with the largest SCFA differences observed in beet pulp tubes (3549 vs. 2980 μmole/g) and the greatest pH differences in pectin tubes (−1.66 vs. –1.54). Microbiota analysis revealed increased relative abundances of SCFA-producing genera, along with reduced relative abundances of *Fusobacterium*, *Streptococcus*, and *Sutterella*. Overall, dried brewers yeast supplementation can influence canine gastrointestinal microbiota composition and fermentative activity in vitro.

## Simple Summary

Dried brewers yeast is increasingly explored as a functional ingredient in canine diets, though product variability may lead to differing outcomes. This experiment evaluated the in vitro fermentation characteristics of different fibers using fecal inocula from dogs fed diets supplemented with dried brewers yeast. Fecal inoculum for the in vitro fermentation assay was obtained from healthy adult dogs fed either a control diet without yeast (CTRL) or a dried brewers yeast-containing diet (BY). Beet pulp, pectin, and cellulose were incubated for 0, 6, 12, or 18 h, with measurements of pH, short-chain fatty acid (SCFA) production, and microbiota evaluated at each time-point. Fermentation characteristics and microbial populations differed by fiber and inoculum source. Pectin was the most fermentable, beet pulp had moderate fermentability, and cellulose had minimal fermentation based on SCFA production and pH changes. At 18 h, BY inocula yielded more SCFA and lower pH than CTRL, with the largest SCFA difference in beet pulp (3549 vs. 2980 μmol/g) and the greatest pH difference in pectin (−1.66 vs. −1.54). Differences in the relative abundances of predominant bacterial genera were also observed between inoculum sources. These results indicate that dried brewers yeast supplementation can change the abundance and activity of canine fecal microbiota in vitro.

## 1. Introduction

With a greater focus on pet health and wellbeing, the pet food industry has increased its focus on functional ingredients that support health beyond basic nutrition [1]. Among these, yeast and yeast-derived ingredients have gained attention due to their high nutritional value and the potential functional attributes of yeast constituents [2]. Yeast-based ingredients contain bioactive compounds, such as polyphenols, mannanoligosaccharides, and β-glucans [3,4]. Some bioactive compounds are absorbed in the small intestine, but a considerable proportion persist into the colon and fuel microbial fermentation, thereby influencing gut microbiota composition and activity [5].

Supplementation with yeast-derived ingredients can modulate gut microbiota and enhance gut health and immunity. Using an in vitro colonic fermentation model, Van Den Abbeele et al. [5] demonstrated that yeast formulations enhanced short-chain fatty acid (SCFA) production and inhibited opportunistic pathogen growth. The yeast products included in that experiment consisted of diverse yeast cell fractions, including purified cell wall components, cytoplasmic extracts, and combinations thereof. Lin et al. [6] reported beneficial microbiota shifts and reduced inflammatory markers in dogs supplemented with *Saccharomyces cerevisiae* fermentation products. However, variations in yeast-derived products, influenced by strain, inclusion level, processing conditions, and the different cellular fractions utilized have contributed to inconsistent results [7]. For instance, supplementation with yeast-enriched functionalized canola meal did not affect stool quality, fecal metabolite profiles, microbiota composition, or immune responses in adult dogs [8,9]. Likewise, Rummell et al. [10] observed no significant differences in fecal SCFA concentrations after supplementing dogs with a concentrated brewers yeast product at a daily β-glucan inclusion of 7 mg/kg of body weight.

Although in vivo experiments are ultimately necessary to confirm the effects and optimal inclusion levels of functional ingredients in the animal species of interest, in vitro fermentation systems serve as a practical and cost-effective approach for initial evaluation of gut microbiota responses [11]. These systems are designed to replicate the microbial dynamics of the gastrointestinal tract, allowing repeated sampling with high reproducibility and without the ethical constraints of in vivo experiments [12]. Additionally, rapid absorption of SCFA in the gut limits the extent to which fecal samples can be used as proxy for microbial activity. In contrast, in vitro fermentation systems allow monitoring of microbiota composition and activity, including fermentation metabolites and pH dynamics. However, their ability to predict in vivo responses is strongly influenced by the system design and the extent to which it replicates in vivo conditions. Batch culture systems, such as the one used in this experiment, are restricted to short incubation times (24–48 h) due to nutrient depletion and accumulation of fermentation end-products [11]. Furthermore, the extent to which results reflect in vivo responses also depends on the inoculum source and the degree to which preserved samples retain the original microbial community, factors that may be influenced by storage conditions and pooling, among others [13,14].

Accordingly, the objective of this experiment was to evaluate the in vitro fermentation patterns of common dietary fibers using fecal inoculum from dogs fed either a control diet or a diet supplemented with a dried brewers yeast product. We hypothesized that tubes inoculated with feces from dogs fed the brewers yeast-supplemented diet would exhibit higher fermentation activity, as indicated by greater SCFA production, and a distinct microbiota composition relative to those inoculated with feces from dogs fed the control diet.

## 2. Materials and Methods

### 2.1. Substrates and Analysis

The dietary fibers used included beet pulp (TDF: 93.0%; Archer Daniels Midland Co., Decatur, IL, USA), high methoxy pectin (TDF: 67.1%; Modernist Pantry LLC; Eliot, ME, USA), and cellulose (TDF: 100.0%; Solka Floc, International Fiber Corporation; North Tonawanda, NY, USA). Cellulose served as the negative control and pectin as the positive control, based on their known fermentability profiles. All fibers were analyzed for dry matter and ash [ref. [15]; methods 934.01 and 942.05] with organic matter calculated by difference. Fibers were analyzed in duplicate, with a deviation of less than 5% between duplicates regarded as acceptable.

### 2.2. Animals, Experimental Design and Sample Collection

Sixteen healthy adult beagles (body weight = 9.01 ± 1.62 kg; age = 4.2 ± 1.8 yr; sex = 7 males, 9 female) of ideal body condition score (3 on a 5-point scale) were used in a completely randomized design. Dogs were individually housed in an environmentally controlled room maintained on a 12 h light/dark cycle. Dogs had free access to fresh water at all times via an automatic watering system. They were fed once daily based on the estimated energy requirements of neutered adult dogs calculated as: 70 × (kg body weight)^0.75^.

The experiment consisted of an acclimation phase (7 d) and an experimental phase (21 d). During acclimation, all dogs were fed a commercial extruded dry diet (Purina Dog Chow; Nestlé Purina PetCare Company, St. Louis, MO, USA). After the acclimation phase, dogs were randomly assigned to receive either a basal control diet without yeast (CTRL; *n* = 8) or the same diet supplemented with 1.5% dried brewer’s yeast (BY; *n* = 8). Both treatment diets were formulated to meet all the essential nutrients required for maintenance of adult dogs, in accordance with the recommendations of the Association of American Feed Control Officials [16]. Treatment diets were coded to ensure researcher blinding throughout the experiment. On the last day of the feeding trial, fresh fecal samples were collected from each dog at the time of its first defecation and processed within 15 min of voiding. Samples were obtained from the dogs’ first defecation during the assigned fecal collection period. For each dog, 10 g of feces was preserved with 10 mL of 20% glycerol in a 50 mL conical tube. Samples were frozen at −80 °C in accordance with the method of Cammarota et al. [17] and shipped overnight on dry ice to the University of Illinois Urbana-Champaign for the in vitro fermentation assay.

### 2.3. In Vitro Fermentation Assay

The in vitro fermentation assay was performed as described in the methods of Bourquin et al. [18]. All media constituents, except the vitamin solutions, were mixed and autoclaved (Table 1). Filter-sterilized vitamin solutions were added prior to medium dispensing. Medium aliquots (26 mL) were aseptically transferred into tubes (50 mL) containing 300 mg of substrate. Blank tubes without substrate were prepared to correct values for baseline microbial activity. To ensure anaerobic conditions, the tubes were sealed with rubber stoppers fitted with unidirectional gas release valves. Tubes were stored at 4 °C for approximately 12 h prior to inoculation to allow substrate hydration. Tubes were transferred to a 39 °C water bath approximately 30 min prior to inoculation to allow for thermal equilibration.

Fecal aliquots were thawed on the morning of the assay using a water bath set to 39 °C. To prepare the fecal inoculum, thawed fecal aliquots were pooled by treatment and diluted 1:4 (wt/vol) in anaerobic diluting solution, then homogenized using a commercial blender (model 31BL41, Waring Commercial, Stamford, CT, USA) for 15 sec under CO_2_. Fecal samples were pooled to acquire the necessary inoculum volume and to reduce variability among replicates within each treatment. The homogeneous mixtures were filtered through 4 layers of cheesecloth, then transferred into 125 mL serum bottles and sealed under a CO_2_ stream to limit oxygen exposure. Both blank and sample tubes were inoculated with 4 mL of fecal inoculum and incubated at 39 °C for 0, 6, 12, and 18 h, with periodic mixing to simulate gut motility. Triplicate tubes were prepared for each fiber × inoculum × time combination, along with corresponding triplicate blanks, for a total of 96 fermentation tubes.

At each time-point, the corresponding set of tubes were retrieved from the incubator and immediately processed for analysis. Briefly, fermentation media pH was measured using a pH meter (Accumet model AB15, Fisher Scientific Inc., Pittsburgh, PA, USA). For SCFA analysis, 2 mL aliquots were mixed with 0.5 mL of 25% metaphosphoric acid and analyzed according to the method of Erwin et al. [19] using a Hewlett–Packard Model 5890A gas chromatograph (model 5890A; Hewlett–Packard, Avondale, PA, USA) equipped with a flame ionization detector and a column (1.8 m × 4 mm i.d.) packed with GP 10% SP-1200/1% H_3_PO_4_ on 80/100 Chromosorb W-AW (Supelco, Bellefonte, PA, USA). Nitrogen was used as the carrier gas at a flow rate of 75 mL/min. The oven, injector, and detector ports were maintained at 125, 175, and 180 °C, respectively. Samples for microbial evaluation were placed in sterile cryogenic tubes, snap frozen on dry ice, and stored at −80 °C.

### 2.4. Microbiota Analysis

Genomic DNA was isolated with the PowerSoil Kit (MO BIO Laboratories, Inc., Carlsbad, CA, USA) following the manufacturer’s instructions. The quality of genomic DNA was evaluated by electrophoresis on a 1% E-Gel EX (Invitrogen, Carlsbad, CA, USA), and concentrations were quantified with a Qubit 3.0 Fluorometer (Life Technologies, Grand Island, NY, USA) using the Qubit dsDNA BR assay kit (Invitrogen, Carlsbad, CA, USA). 16S rRNA gene amplicons were generated using the Fluidigm Access Array (Fluidigm Corporation, South San Francisco, CA, USA) in combination with the Roche High Fidelity Fast Start Kit (Roche, Indianapolis, IN, USA). Amplification of a 252 bp fragment within the V4 region of the 16S rRNA gene was performed using primers (IDT Corp., Coralville, IA, USA) 515F (5′-GTGCCAGCMGCCGCGGTAA-3′) and 806R (5′-GGACTACHVGGGTWTCTAAT-3′), as described by Caporaso et al. [20]. Forward CS1 and reverse CS2 tags were added following to the Fluidigm protocol. Amplicon quality was assessed with a Fragment Analyzer (Advanced Analytics, Ames, IA, USA) to verify both size and target region. A DNA pool was prepared by combining equimolar amounts of the amplicons from all samples. The pooled DNA was then size-selected on a 2% agarose E-gel (Life Technologies, Grand Island, NY, USA) and extracted using a Qiagen gel purification kit (Qiagen, Valencia, CA, USA). After cleanup and size selection, pooled products were run on an Agilent Bioanalyzer to confirm proper fragment distribution and average length. Sequencing was performed on an Illumina MiSeq using v3 reagents (Illumina Inc., San Diego, CA, USA) at the Roy J. Carver Biotechnology Center at the University of Illinois Urbana-Champaign.

### 2.5. Bioinformatics

Forward reads were trimmed using the FASTX-Toolkit (version 0.0.13), and the resulting sequences were processed with the QIIME 2.0 software pipeline [21]. Sequences with a quality score ≥ 20 were filtered using the Divisive Amplicon Denoising Algorithm (DADA2) pipeline for quality control [22]. Samples were rarefied to a minimum depth of 25,200 reads, the point at which diversity curves plateaued. Taxonomy was assigned using the QIIME2 classifier trained on the Silva 138 reference database at a 99% similarity threshold, specifically targeting the 515F/806R V4 region of the 16S gene [23,24,25]. Diversity analyses were conducted via the q2-diversity plugin. Alpha diversity was assessed using the q2-diversity plugin with following metrics: number of observed features, Faith’s phylogenetic diversity [26], and the Shannon diversity index [27]. Beta diversity was calculated using weighted and unweighted UniFrac distances [28] and visualized with principal coordinate analysis (PCoA) plots.

### 2.6. Statistical Analyses

Blank corrected data were analyzed using the GLIMMIX procedure of SAS 9.4 (SAS Institute Inc., Cary, NC, USA). An initial analysis was conducted considering the main effects of fiber source, time, and the fiber × time interactions. Subsequently, within each fiber substrate, the effects of treatment, time, and treatment × time interactions were assessed. Statistical differences were determined using a Fisher-protected least significant difference (LSD) test with a Tukey adjustment to control for experiment-wise error. Statistical significance was declared as *p* ≤ 0.05.

## 3. Results

### 3.1. Differences Between Fiber Substrates

**Figure 1:**
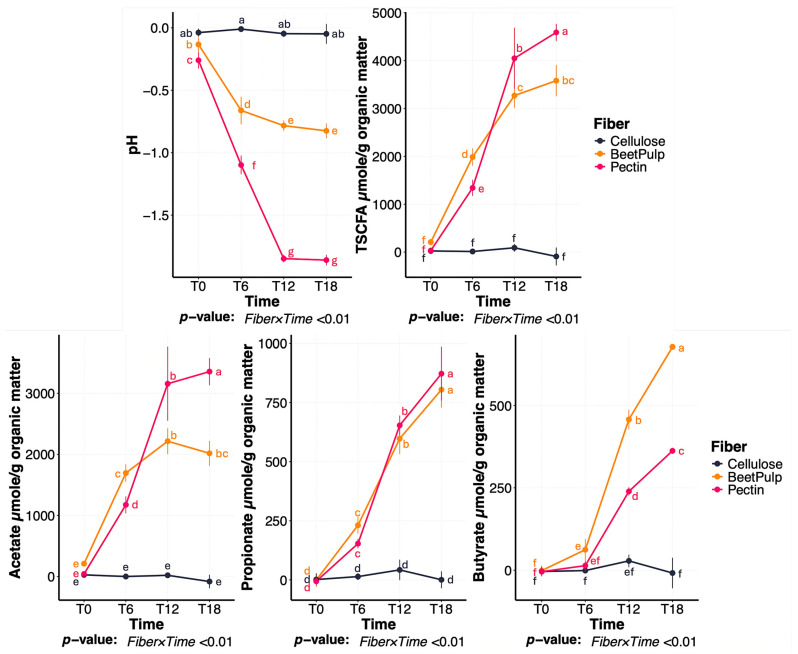
Change in fermentation media pH and short-chain fatty acid (SCFA) concentrations during in vitro fermentation of cellulose, beet pulp, and pectin using fecal inocula from dogs. ^a–g^ Means with different superscript differ (*p* ≤ 0.05). TSCFA = total SCFA, T0 = immediately after inoculation, T6 = 6 h after inoculation, T12 = 12 h after inoculation, T18 = 18 h after inoculation.

A significant (*p* < 0.01) fiber×time interaction was observed for fermentation media pH and SCFA production (Figure 1; Supplementary Table S1). Over time, media pH decreased and SCFA production increased, with differences noted across fiber sources. Specifically, pectin had the greatest (*p* < 0.01) pH reduction and SCFA production followed by beet pulp and cellulose. Likewise, acetate production was greatest (*p* < 0.01) in pectin tubes, followed by beet pulp and cellulose, with all fibers being different from one another. Propionate production was greater (*p* < 0.01) in tubes containing pectin and beet pulp than those containing cellulose. For butyrate production, tubes containing beet pulp had a greater (*p* < 0.01) increase than all other tubes. Butyrate production was also greater (*p* < 0.01) in tubes containing pectin than those containing cellulose.

**Figure 2:**
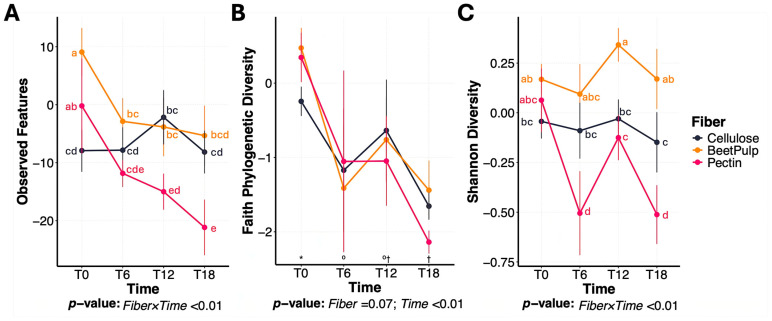
Change in alpha diversity measures during in vitro fermentation of cellulose, beet pulp, and pectin using fecal inocula from dogs. (**A**) Observed features, (**B**) Faith phylogenetic diversity, and (**C**) Shannon Diversity Index illustrating changes in alpha diversity across fiber substrates over time. ^a–e^ Means with different superscript differ (*p* ≤ 0.05). T0 = immediately after inoculation, T6 = 6 h after inoculation, T12 = 12 h after inoculation, T18 = 18 h after inoculation.

**Figure 3:**
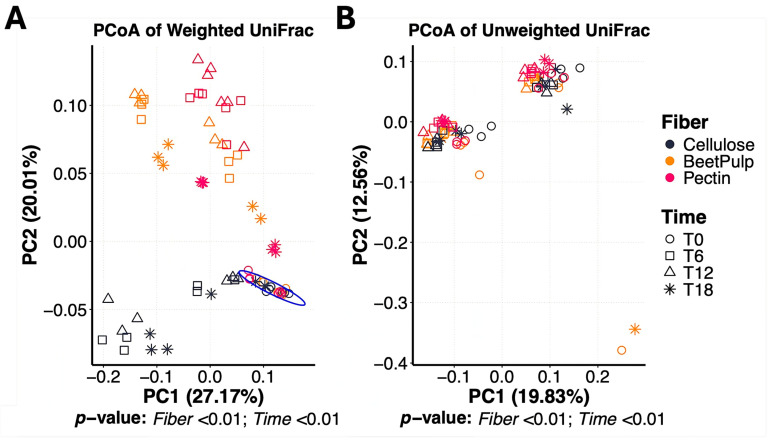
Bacterial beta diversity measures of fermentation media during cellulose, beet pulp, and pectin in vitro fermentation assays using fecal inocula from dogs. Principal coordinates analysis (PCoA) plots of weighted (**A**) and unweighted (**B**) UniFrac distances revealed three distinct clusters corresponding to each fiber source, as well as two clusters based on time, with T0 separating from all subsequent time-points. T0 = immediately after inoculation, T6 = 6 h after inoculation, T12 = 12 h after inoculation, T18 = 18 h after inoculation.

Bacterial alpha diversity measures, namely observed features and Shannon Diversity Index, were affected (*p* < 0.01) by a fiber × time interaction (Figure 2). Over time, the number of observed features was greater (*p* < 0.01) in beet pulp and cellulose tubes than those containing pectin. Shannon Diversity Index was greater (*p* < 0.01) in beet pulp tubes than all other fibers, and greater in cellulose tubes than those containing pectin. Faith’s phylogenetic diversity decreased (*p* < 0.01) over time, with no differences (*p* = 0.07) among fibers. PCoA plots of weighted and unweighted UniFrac distances representing bacterial beta diversity measures between fibers are shown in Figure 3. In this experiment, the bacterial community at 0 h (immediately after inoculation) was different (pseudo-F = 3.26; *p* < 0.01) from that at 6, 12 or 18 h. Microbiota composition also differed among fiber substrates (pseudo-F = 7.71; *p* < 0.01). Given the distinct fermentation dynamics and microbiota profiles observed among fibers, treatment effects (BY vs. CTRL) were evaluated within each fiber.

### 3.2. Change Between Treatments During Beet Pulp Fermentation

**Figure 4:**
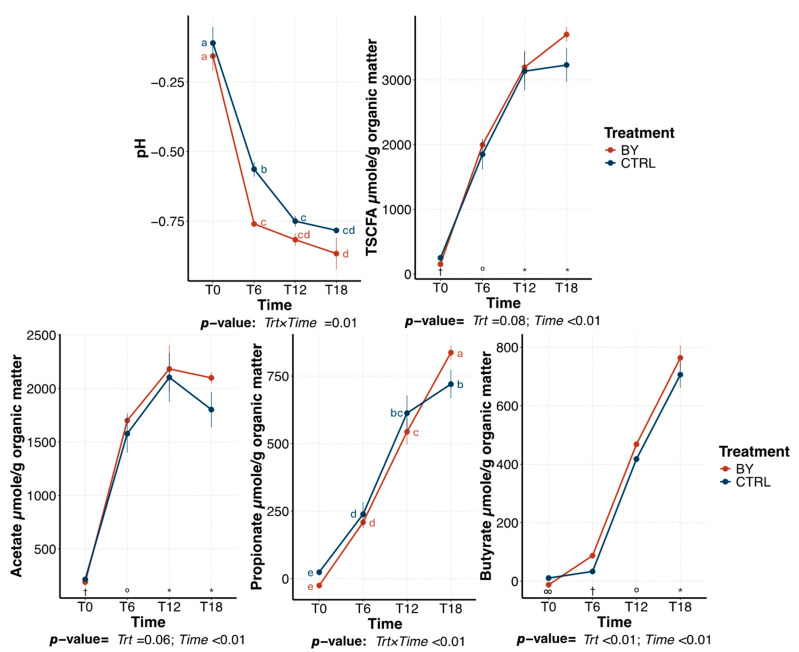
Change in pH and short-chain fatty acid (SCFA) concentrations during a beet pulp fermentation assay using fecal inocula from dogs fed the control diet (CTRL) or the dried brewers yeast diet (BY). ^a–e,^*^,°,†,∞^Means with different superscript differ (*p* ≤ 0.05). TSCFA = total SCFA, T0 = immediately after inoculation, T6 = 6 h after inoculation, T12 = 12 h after inoculation, T18 = 18 h after inoculation.

Within beet pulp tubes, a treatment × time interaction was observed for pH, propionate production, and butyrate production (Figure 4). Fermentation media pH decreased over time, with BY tubes exhibiting a greater decrease than those inoculated with CTRL (Δ0/18: −0.67 vs. −0.71). Total SCFA and acetate increased over time (*p* < 0.01), with no significant differences between treatments. However, tubes inoculated with BY had a greater increase (*p* < 0.01) in propionate (Δ0/18: 696 vs. 862 μmole/g) and butyrate (696 vs. 777 μmole/g) production over time than tubes inoculated with CTRL.

**Figure 5:**
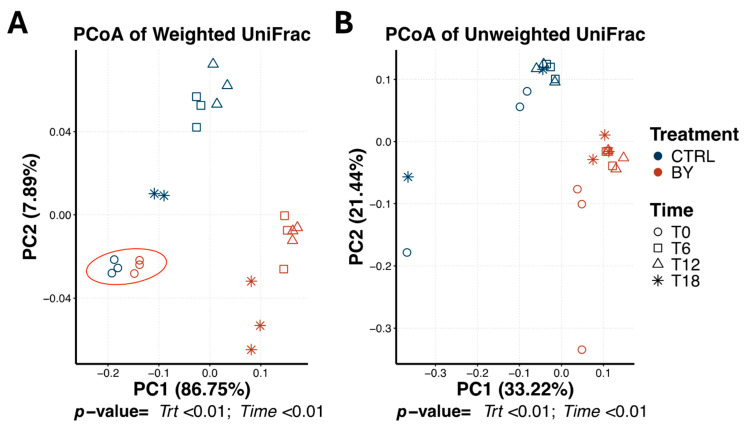
Bacterial beta diversity measures of fermentation media during beet pulp in vitro fermentation assay using fecal inocula from dogs fed the control diet (CTRL) or diet containing dried brewers yeast (BY). Principal coordinates analysis (PCoA) plots of weighted (**A**) and unweighted (**B**) UniFrac distances revealed two distinct clusters corresponding to each dietary treatment. For each treatment, samples collected at T0 (immediately after inoculation) are clustered separately from those collected at 12 and 18 h, while samples collected at 6 h do not show a distinct separation from any other time. T0 = immediately after inoculation, T6 = 6 h after inoculation, T12 = 12 h after inoculation, T18 = 18 h after inoculation.

Bacterial beta diversity within beet pulp tubes differed by treatment and time (Treatment: pseudo-F = 8.97 [weighted], 7.65 [unweighted]; Time: pseudo-F = 1.61 [weighted], 1.61 [unweighted]; *p* < 0.01; Figure 5). Bacterial communities were primarily separated by treatment, with further temporal differences evident between 0 h and those at 12 and 18 h. The bacterial community at 6 h was not different from any other time-point. Based on 16S rRNA gene sequencing, the microbial profile of the in vitro fermentation media within beet pulp tubes was dominated by the Firmicutes (71.72% of sequences), Bacteroidota (12.85% of sequences), Fusobacteriota (9.81% of sequences), Actinobacteriota (3.74% of sequences) and Proteobacteria (1.86% of sequences) phyla.

**Figure 6:**
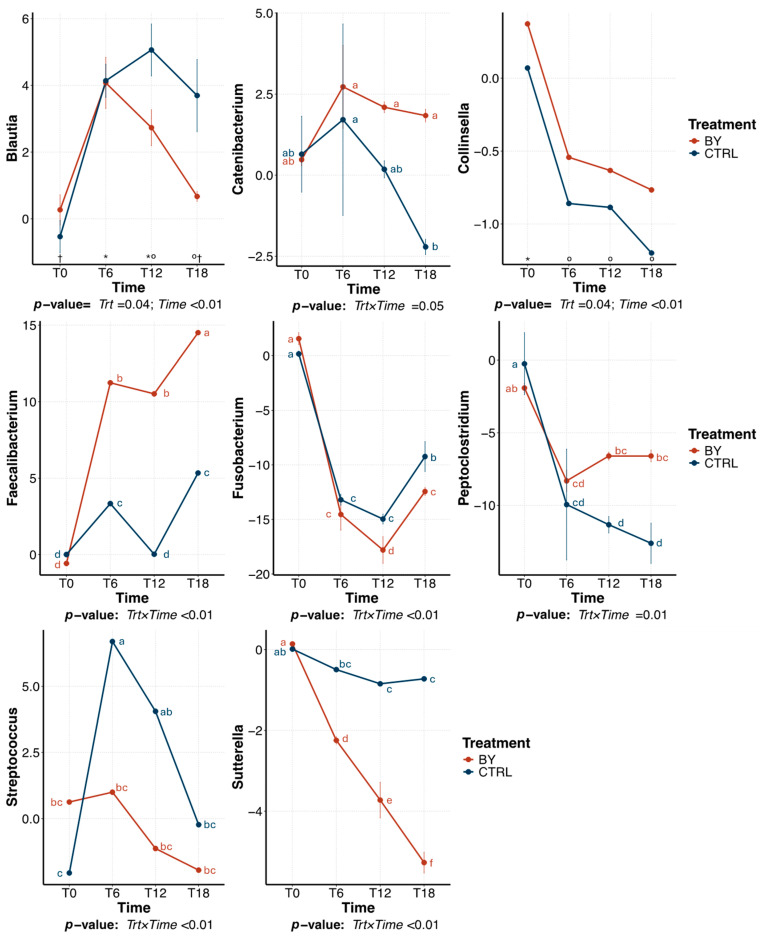
Change in relative abundances (% of sequences) of predominant bacterial genera during beet pulp fermentation with fecal inoculum from dogs fed the control diet (CTRL) or diet containing dried brewers yeast (BY). ^a–f,^*^–†^ Means with different superscript differ (*p* ≤ 0.05). T0 = immediately after inoculation, T6 = 6 h after inoculation, T12 = 12 h after inoculation, T18 = 18 h after inoculation.

The predominant bacterial genera (relative abundance > 1% of sequences) that were significantly affected by treatment within beet pulp tubes are presented in Figure 6. The relative abundance of *Blautia* was highest at 12 h of fermentation and declined thereafter, with a greater reduction in tubes inoculated with CTRL than those inoculated with BY (*p* = 0.04). The relative abundance of *Collinsella* was highest (*p* < 0.01) at 0 h and decreased over time. Several other genera were affected (*p* < 0.01) by a treatment×time interaction. Over time, the relative abundance of *Catenibacterium* decreased (*p* = 0.05) in CTRL tubes, but remained stable in BY. *Faecalibacterium* relative abundance increased (*p* < 0.01) in both treatments, but more so in BY tubes (*p* < 0.01). *Fusobacterium* relative abundance decreased (*p* < 0.01) over time in both treatments, with a greater reduction (*p* < 0.01) in BY tubes. In contrast, *Peptoclostridium* had a greater (*p* = 0.01) reduction in CTRL than in BY tubes. *Streptococcus* had a greater increase (*p* < 0.01) in BY tubes than CTRL tubes. *Sutterella* decreased (*p* < 0.01) in both treatments, but more so in BY tubes.

### 3.3. Change Between Treatments During Pectin Fermentation

**Figure 7:**
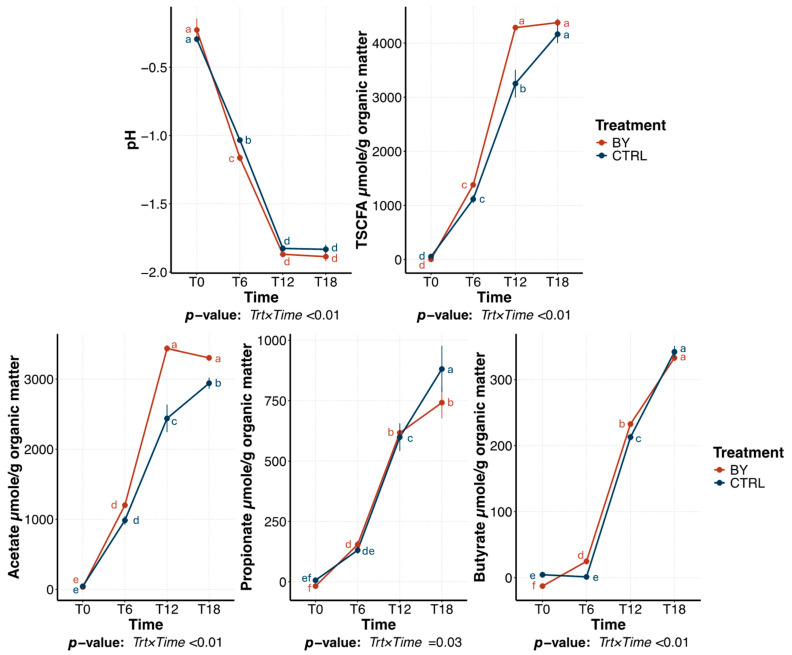
Change in pH and short-chain fatty acid (SCFA) concentrations during pectin fermentation assay using fecal inocula from dogs fed the control diet (CTRL) or the dried brewers yeast diet (BY). ^a–f^ Means with different superscript differ (*p* ≤ 0.05). TSCFA = total SCFA, T0 = immediately after inoculation, T6 = 6 h after inoculation, T12 = 12 h after inoculation, T18 = 18 h after inoculation.

The fermentation profile of pectin tubes followed similar patterns to those observed with beet pulp. Briefly, fermentation media pH and SCFA production were affected (*p* < 0.01) by a treatment × time interaction. Fermentation media pH decreased (*p* < 0.01) over time, but tubes inoculated with BY had a greater (*p* < 0.01) reduction than tubes inoculated with CTRL (−1.54 vs. 1.66, respectively; Figure 7). SCFA production increased (*p* < 0.01) in both treatments over time. Specifically, total SCFA concentrations were greater (*p* < 0.01) in BY tubes than CTRL at 12 h, but no differences were observed between treatments at 18 h. Acetate production was also greater in BY tubes than CTRL tubes. Propionate production was greater (*p* = 0.03) in CTRL tubes, while butyrate was greater in BY tubes at 6 and 12 h (*p* < 0.01), with no difference between treatments at 18 h.

**Figure 8:**
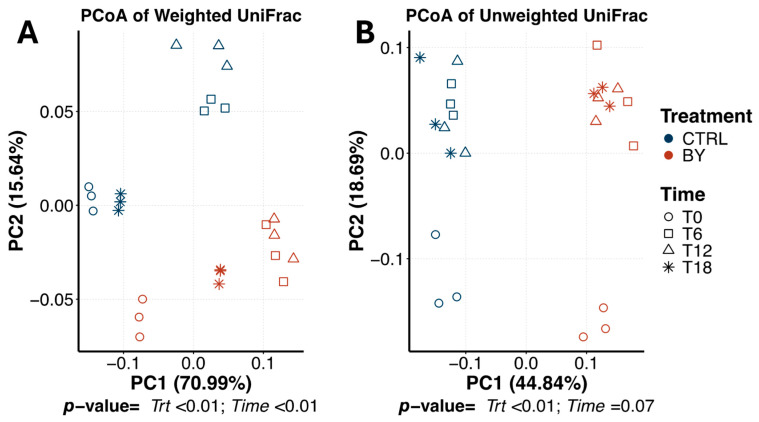
Bacterial beta diversity measures of fermentation media during pectin in vitro fermentation assay using fecal inocula from dogs fed the control diet (CTRL) or diet containing dried brewers yeast (BY). Principal coordinates analysis (PCoA) plots of weighted (**A**) and unweighted (**B**) UniFrac distances revealed two distinct clusters corresponding to each dietary treatment. Based on weighted UniFrac distances, samples also clustered distinctly by time. T0 = immediately after inoculation, T6 = 6 h after inoculation, T12 = 12 h after inoculation, T18 = 18 h after inoculation.

Bacterial beta diversity within pectin tubes is shown in Figure 8. PCoA plots based on weighted and unweighted UniFrac distances showed distinct (*p* < 0.01) clusters by treatment (pseudo-F = 10.31 [weighted], 17.56 [unweighted]). Weighted UniFrac distances also showed temporal separation, with 0 h and 6 h communities clustering apart from those at 12 and 18 h (pseudo-F = 10.41; *p* < 0.01). No time effect was observed based on the unweighted UniFrac distances (pseudo-F = 1.76; *p* = 0.07).

**Figure 9:**
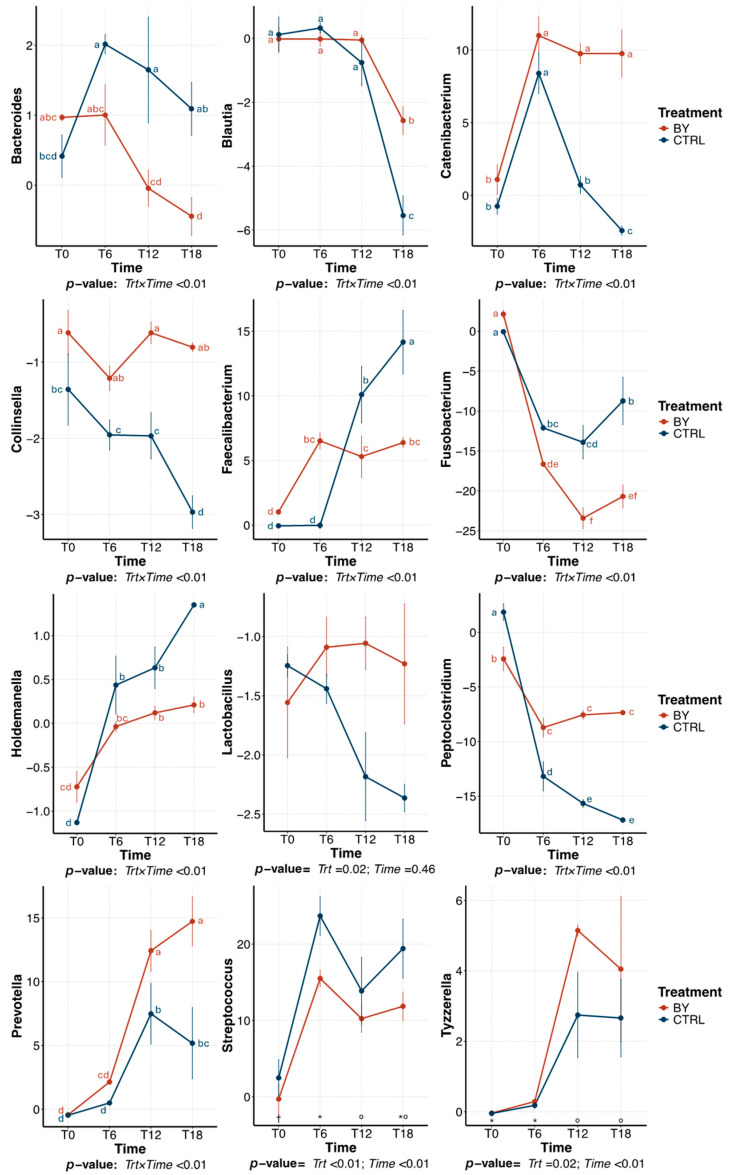
Change in relative abundances (% of sequences) of predominant bacterial genera during pectin fermentation with fecal inoculum from dogs fed the control diet (CTRL) or diet containing dried brewers yeast (BY). ^a–f,^ Means with different superscript differ (*p* ≤ 0.05). T0 = immediately after inoculation, T6 = 6 h after inoculation, T12 = 12 h after inoculation, T18 = 18 h after inoculation.

The bacterial community within pectin tubes was dominated by Firmicutes (78.75 ± 10.23% of sequences), Bacteroidota (9.65 ± 6.52% of sequences), Fusobacteriota (8.19 ± 5.82% of sequences), Actinobacteriota (2.46 ± 1.46% of sequences) and Proteobacteria (0.94 ± 0.52% of sequences). Treatment effects on predominant bacterial genera within pectin tubes are illustrated in Figure 9. Specifically, BY tubes had a greater (*p* = 0.02) increase in *Lactobacillus* while *Streptococcus* abundance was greater (*p* < 0.01) in CTRL tubes. The relative abundance of *Tyzerella* was highest (*p* < 0.01) at 12 h and stabilized thereafter, but BY tubes had a greater (*p* = 0.02) abundance than CTRL tubes. Other predominant bacterial genera identified were affected (*p* < 0.01) by a treatment×time interaction. Briefly, *Collinsella* decreased (*p* < 0.01) in CTRL tubes, but did not change in BY tubes. *Bacteroides* decreased (*p* < 0.01) over time in BY tubes but increased in CTRL tubes. *Prevotella* abundance increased (*p* < 0.01) in both treatments, but was higher in BY tubes. *Blautia* decreased (*p* < 0.01) in both treatments, but was lower in tubes inoculated with CTRL. The relative abundance of *Catenibacterium* increased (*p* < 0.01) in BY tubes after 6 h, but decreased in CTRL tubes. *Faecalibacterium* and *Holdemanella* increased (*p* < 0.01) over time, but CTRL tubes showed a greater increase. *Peptoclostridium* decreased (*p* < 0.01) in both treatments, but more so in CTRL tubes. Lastly, the relative abundance of *Fusobacterium* decreased (*p* < 0.01) over time but was lowest in tubes inoculated with BY than those inoculated with CTRL.

### 3.4. Change Between Treatments During Cellulose Fermentation

Changes in pH and SCFA concentrations during cellulose fermentation are shown in Supplementary Figure S1. Fermentation media pH was not affected by treatment (*p* = 0.53) or time (*p* = 0.43). However, a significant (*p* < 0.01) treatment×time interaction was observed for SCFA production. Total SCFA (Δ12/18 h: −78.3), acetate (Δ12/18 h: −120.6) and butyrate (Δ12/18 h: 3.6) concentrations decreased in BY tubes after 18 h (*p* < 0.01), but did not change in CTRL tubes. Propionate production increased (*p* < 0.01) after 6 h and stabilized thereafter.

PCoA plots based on weighted and unweighted UniFrac distances showed distinct clustering by treatment (weighted: pseudo-F = 14.84, *p* < 0.01; unweighted: pseudo-F = 15.24, *p* < 0.01), but not by time (weighted: pseudo-F = 1.56; unweighted: pseudo-F = 1.56; *p* > 0.09; Supplementary Figure S2). The bacterial community within cellulose tubes was largely dominated by the Firmicutes (71.25%), Fusobacteriota (18.45%), Actinobacteriota (4.51%), Proteobacteria (2.77%), and Bacteroidota (3.02%). Predominant genera affected by treatment during cellulose fermentation are presented in Supplementary Figure S3. Cellulose tubes inoculated with BY had lower abundance of *Blautia* (*p =* 0.04), *Collinsella* (*p =* 0.04), and *Sutterella* (*p =* 0.01) compared to CTRL, and a greater increase (*p =* 0.04) in *Fusobacterium*.

## 4. Discussion

As expected, distinct fermentation patterns were observed among the fiber substrates evaluated. These differences reflect the structural characteristics and enzymatic accessibility inherent to each substrate. High methoxyl pectin, a soluble fiber found in primary plant cell walls, is commonly used in pet food as a fiber supplement and gelling agent [29,30,31]. This dietary fiber was selected as a positive fermentation control because of its well-documented high fermentability [32,33]. Beet pulp, a byproduct of sugar beet processing, is a moderately fermentable fiber that consists of both soluble and insoluble fiber components in a desirable ratio [34,35]. Lastly, Solka-Floc, a purified cellulose fiber, served as a negative fermentation control due to its limited fermentability by canine gut microbiota [36,37].

In vitro fermentation of the different fibers increased SCFA production and altered media pH, with the rate and extent of these changes varying by substrate. Tubes containing pectin showed the greatest decrease in pH and the highest SCFA production, while beet pulp elicited a moderate response. In contrast, tubes containing cellulose had minimal SCFA production and maintained stable pH levels over time. These results are consistent with findings from Sunvold et al. [38], which evaluated the in vitro fermentability of different fibrous substrates using canine fecal inoculum over a 24 h period. In that experiment, highly fermentable substrates had the highest SCFA production and organic matter disappearance, while cellulose-rich substrates exhibited minimal fermentation.

Because advanced analytical methods were previously unavailable, earlier in vitro experiments could not evaluate the effects of different fiber sources on microbiota composition and dynamics. In the current experiment, 16S rRNA gene amplicon sequencing was used to assess the temporal changes in bacterial community composition during the in vitro fermentation of pectin, beet pulp, and cellulose. This high-throughput approach enables dynamic sampling and provides high sensitivity to taxonomic changes, allowing for a more detailed analysis of the complex bacterial community within the in vitro model [39,40].

Based on alpha diversity measures, bacterial richness and diversity decreased over time. This decline, however, was more pronounced in pectin tubes than those containing beet pulp, while no significant changes were noted in cellulose tubes. A recent study from our laboratory evaluating the in vitro fermentation of acacia fiber, pectin, inulin, and cellulose [41] reported similar declines during pectin and inulin fermentation. These findings suggest that pectin may favor the proliferation of a few specific bacterial taxa, leading to reduced overall diversity, whereas beet pulp may support a broader range of microbes and help maintain community diversity over time.

Beta diversity analyses in the current experiment demonstrated differences in bacterial community structure among fiber substrates, illustrated by distinct clusters in the PCoA plots. Previous studies have shown that substrate availability shapes microbial composition by providing distinct ecological niches and energy sources [42]. This concept has been well studied as it applies to both humans and companion animals [43,44,45,46,47].

Given the differences observed among substrates, the effects of treatment were evaluated within each fiber source; however, discussion is centered on general patterns consistent among substrates. Yeast-derived products are commonly used in the pet food industry as functional ingredients, though their composition varies based on source and processing. In the current experiment, a few common dietary fiber sources were selected to evaluate the effects of supplementing a dried brewers yeast product on microbiota community dynamics in vitro.

Fermentation media pH differed between treatments, with tubes inoculated with BY having a larger pH reduction than those inoculated with CTRL. This was accompanied by higher SCFA production in BY tubes, suggesting increased microbial activity and substrate utilization. However, recent in vivo experiments testing yeast-based products have not shown significant differences in fecal SCFA concentrations [8,9,10,48]. In vivo, SCFA are primarily absorbed by colonic epithelial cells and metabolized in various tissues, with only a small fraction (5–10%) being excreted in the feces [49]. As a result, fecal SCFA concentrations may not directly reflect microbial activity, which could potentially explain the lack of observed differences in those experiments. Therefore, although in vitro models provide useful information into fermentative responses, they do not account for host absorption and metabolism, and thus the magnitude of observed differences may not directly translate to in vivo outcomes.

Tubes inoculated with BY also showed increased relative abundances of several bacterial genera associated with carbohydrate fermentation and SCFA production, though some responses varied by fiber source. Specifically, tubes inoculated with BY had a greater relative abundance of *Catenibacterium* and *Collinsella* than tubes inoculated with CTRL. *Catenibacterium* is a polysaccharide degrader and member of the Firmicutes phyla that has been shown to be increased in dogs consuming a high fiber diet [50] and humans consuming a diet rich in plant-derived foods [51]. *Collinsella* is another polysaccharide degrader, that primarily produces acetate, formate, and lactate during fermentation [52]. This genus has also been found to increase in response to 0.6% dietary yeast culture supplementation in dogs [53]. Tubes inoculated with BY also had greater relative abundances of *Blautia* and *Faecalibacterium*. *Blautia*, a known saccharolytic genus, produces acetate as its main product of fermentation [54]. Dietary supplementation with yeast-purified β-1,3/1,6-glucans at concentrations of 0.07% and 0.28% has been shown to increase *Blautia* abundance in canine feces [55]. *Faecalibacterium* predominantly ferments glucose into formate, small amounts of D-lactate, and significant quantities of butyrate [56,57]. Although fiber source had a strong selective effect on the microbiota in this experiment, a greater abundance of saccharolytic taxa in BY tubes may explain the differences in fermentation activity between inocula.

Tubes inoculated with BY also limited the proliferation of *Fusobacterium*. *Fusobacterium* is a natural member of the canine gut microbiota but is regarded as undesirable due to its role in protein fermentation [43]. This process generates harmful metabolites such as ammonia, amines, phenols, and sulfides, which are detrimental to gut health and linked to various diseases [58]. Consistent with the results of this study, Marchi et al. [55] reported a significant reduction in *Fusobacterium* abundance in the fecal samples of healthy adult dogs supplemented with 0.14% yeast-purified beta-1,3/1,6-glucans. Likewise, greater dietary fiber intake has been shown to decrease the abundance of *Fusobacterium* in fecal samples of healthy adult dogs [59]. In contrast, *Peptoclostridium* relative abundance was increased in tubes inoculated with BY. Like *Fusobacterium*, *Peptoclostridium* is also associated with protein catabolism. The distinct responses of *Fusobacterium* and *Peptoclostridium* to BY inoculation likely stem from differences in substrate preferences and/or microbial interactions within the system, the mechanisms of which remain unclear. Future studies using targeted metabolomics are warranted to evaluate whether observed compositional shifts reflect true changes in proteolytic activity.

Tubes inoculated with BY also had a lower relative abundance of *Streptococcus* than tubes inoculated with CTRL. This genus is frequently elevated in diseased conditions in dogs and is used as a marker of gut microbiota imbalances [60]. Additionally, tubes inoculated with BY had a greater reduction in *Sutterella* than those inoculated with CTRL. *Sutterella* is a bacterial genus within the Proteobacteria phylum known for its role in protein metabolism [61,62]. In a previous experiment, fecal samples from healthy dogs, dogs with acute non-hemorrhagic diarrhea, dogs with acute hemorrhagic diarrhea, and dogs with active or therapeutically controlled idiopathic inflammatory bowel disease were analyzed by 16S rRNA gene sequencing. Dogs with acute hemorrhagic diarrhea had a greater *Sutterella* relative abundance, indicating its potential negative implication on gastrointestinal health [63].

## 5. Conclusions

Beet pulp, pectin and cellulose had distinct and predictable fermentation patterns in vitro. Moreover, the fecal microbial community from dogs consuming a diet supplemented with dried brewers yeast had distinct microbiota composition and fermentation activity in vitro. Specifically, BY inoculated tubes had increased fermentation activity, as indicated by a greater SCFA production and larger pH reductions in vitro. Additionally, BY had increased abundance of SCFA-producing bacteria, such as *Catenibacterium* and *Collinsella*, while inhibiting the proliferation of potentially undesirable bacterial groups, including *Fusobacterium*, *Streptococcus*, and *Sutterella*. However, these findings should be interpreted with caution, as in vitro models do not fully replicate the complexity of the canine gastrointestinal environment. In this regard, validation through in vivo studies, together with the implementation of untargeted metabolomics, could be a key to clarifying the relationship between the microbial shifts observed and functional outputs.

## Figures and Tables

**Table 1:** Composition of medium used for the in vitro fermentation assay.

Component	Concentration in Medium
	---mL/L---
Mineral solution A ^1^	330
Mineral solution B ^2^	330
Trace mineral solution ^3^	10
Water-soluble vitamins ^4^	20
Folate:biotin solution ^5^	5
Riboflavin solution ^6^	5
Hemin solution ^7^	2.5
Short-chain fatty acid mix ^8^	0.4
Resazurin solution ^9^	1.0
Distilled H_2_O	296
	---g/L---
Yeast extract	0.5
Trypticase	0.5
Na_2_CO_3_	4.0
Cysteine HCl·H_2_O	0.5

^1^ Composition (g/L): NaCl, 5.4; KH_2_PO_4_, 2.7; CaCl_2_·H_2_O, 0.18; MgCl·6H_2_O,0.12; MnCl_2_·4H_2_O, 0.06; CoCl_2_·6H_2_O, 0.06; (NH_4_)_2_SO_4_, 5.4. ^2^ Composition: K_2_HPO_4_, 2.7 g/L. ^3^ Composition (g/L): EDTA (disodium salt), 0.5; FeSO_4_·7H_2_O, 0.2; ZnSO_4_·7H_2_O, 0.01; MnCl_2_·4H_2_O, 0.003; H_3_PO_4_, 0.03; CoCl_2_·6H_2_O, 0.02; CuCl_2_·2H_2_O, 0.001; NiCl_2_·6H_2_O, 0.002; Na_2_MoO_4_·2H_2_O, 0.003. ^4^ Composition (g/L): thiamin HCl, 0.1; pantothenic acid, 0.01; niacin, 0.1; pyridoxine, 0.1; p-aminobenzoic acid, 0.005; vitamin B-12, 0.0025. ^5^ Composition (g/L): folic acid, 0.01; biotin, 0.02; NH_4_HCO_3_, 0.1. ^6^ Composition: riboflavin, 10 mg/L in 5 mmol/L HEPES. ^7^ Hemin, 500 mg/L in 10 mmol/L NaOH. ^8^ 250 mL/L each of n-valerate, isovalerate, isobutyrate and DL-a-methylbutyrate. ^9^ Resazurin, 1 g/L in distilled H_2_O. Solution compositions were prepared according to the protocol described by Bourquin et al. [18]. All solutions were brought to volume using distilled water unless otherwise specified.

## Data Availability

The data that support the findings of this experiment are available from the corresponding author upon reasonable request. These data are not publicly available due to their commercial value.

## Supplementary Materials

The following supporting information can be downloaded at: https://www.mdpi.com/article/10.3390/ani15213117/s1, Supplementary Figure S1: Change in pH and short-chain fatty acid (SCFA) concentrations during cellulose fermentation assay using fecal inocula from dogs fed the control diet (CTRL) or the dried brewers yeast diet (BY); Supplementary Figure S2: Bacterial beta diversity measures of fermentation media during cellulose in vitro fermentation assay using fecal inocula from dogs fed the control diet (CTRL) or diet containing dried brewers yeast (BY); Supplementary Figure S3: Change in relative abundances (% of sequences) of predominant bacterial genera during cellulose fermentation with fecal inoculum from dogs fed the control diet (CTRL) or diet containing dried brewers yeast (BY). Supplementary Table S1: Change in short-chain fatty acid concentrations (estimate ± SEM) for each fiber × time, independent of inoculum source.

## Abbreviations

BY: dried brewers yeast, CTRL: control, SCFA: short-chain fatty acids, TSCFA: total short-chain fatty acids
