# Supplementary material for: In Vitro Fermentation Characteristics of Dietary Fibers Using Fecal Inoculum from Dogs Consuming a Dried Brewers Yeast Product
Source: Animals (Basel). 2025 Oct 27;15(21):3117. doi: 10.3390/ani15213117 (PMC12607596; doi:10.3390/ani15213117)
Supplement: Supplementary file 1 [file animals-15-03117-s001.zip › animals-3893835-supplementary.pdf]

Supplementary Table 1. Change in short-chain fatty acid concentrations (estimate ± SEM) for each fiber × time, independent of inoculum source

Fiber	Time	TSCFA (µmole/g)	Acetate (µmole/g)	Propionate (µmole/g)	Butyrate (µmole/g)
Beet pulp	T6	1987.35 ± 72.81	1694.07 ± 57.21	231.11 ± 14.4	62.17 ± 12.93
Beet pulp	T12	3270.6 ± 103.72	2215.52 ± 84.74	597.43 ± 26.34	457.65 ± 11.77
Beet pulp	T18	3581.87 ± 131.31	2017.41 ± 81.88	804.54 ± 30.77	759.92 ± 20.67
Pectin	T6	1341.5 ± 66.47	1174.12 ± 55.34	153.42 ± 7.79	13.96 ± 5.8
Pectin	T12	4049.7 ± 258.49	3156.85 ± 245.72	653.58 ± 16.65	239.28 ± 4.96
Pectin	T18	4590.42 ± 70.7	3355.63 ± 89.64	872.38 ± 46.09	362.41 ± 3.5
Cellulose	T6	12.69 ± 7.6	-0.24 ± 1.46	13.94 ± 5.54	-1.01 ± 2.31
Cellulose	T12	90.32 ± 30.93	19.78 ± 9.19	42.17 ± 17.33	28.36 ± 7.44
Cellulose	T18	-90.49 ± 74.71	-82.51 ± 43.61	0.47 ± 14.33	-8.46 ± 18.65

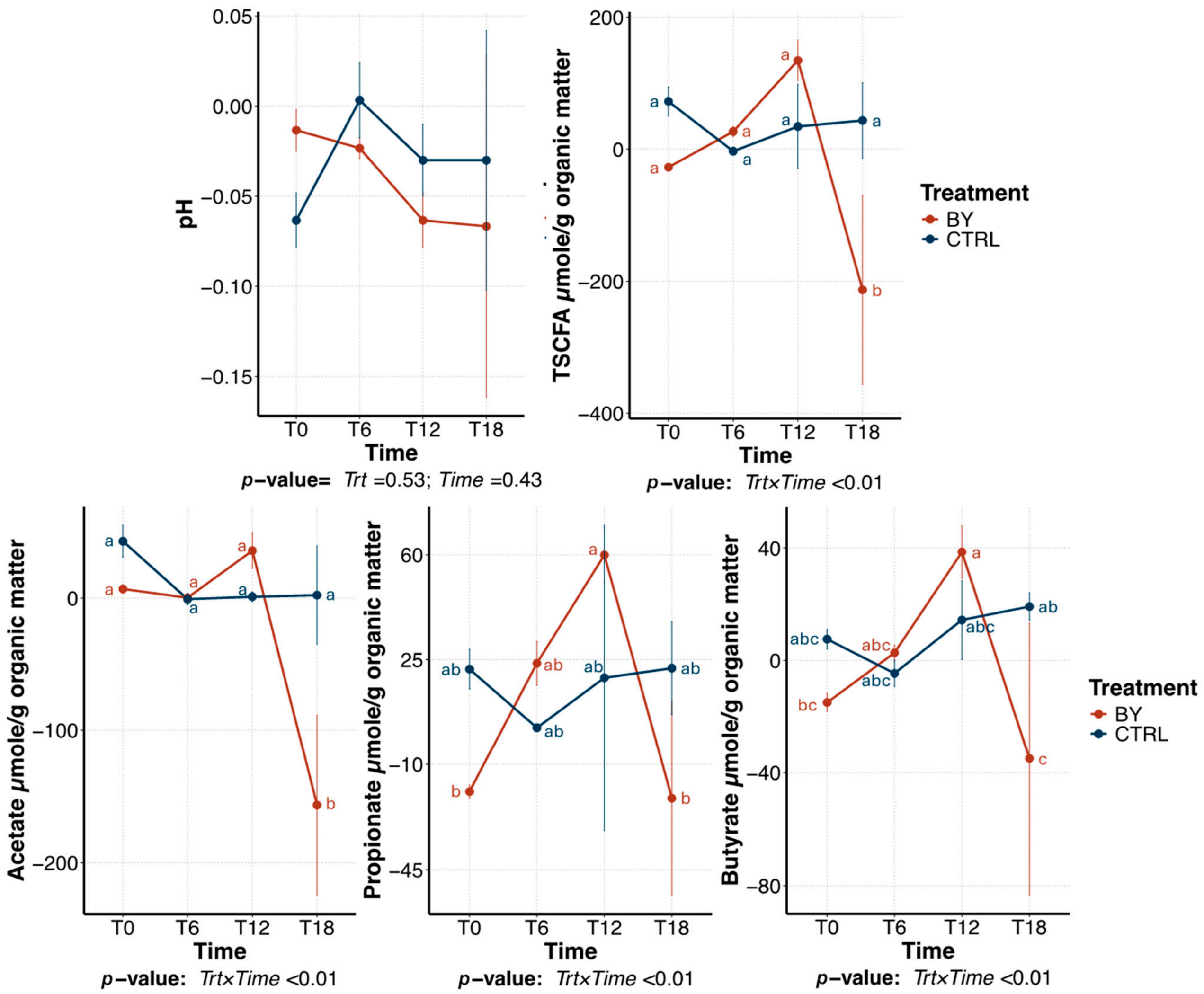

Supplementary Figure 1. Change in pH and short-chain fatty acid (SCFA) concentrations during cellulose fermentation assay using fecal inocula from dogs fed the control diet (CTRL) or the dried brewer's yeast diet (BY). [a-c]Means with different superscript differ ($P \leq 0.05$). TSCFA = total SCFA, T0 = immediately after inoculation, T6 = 6 h after inoculation, T12 = 12 h after inoculation, T18 = 18 h after inoculation.

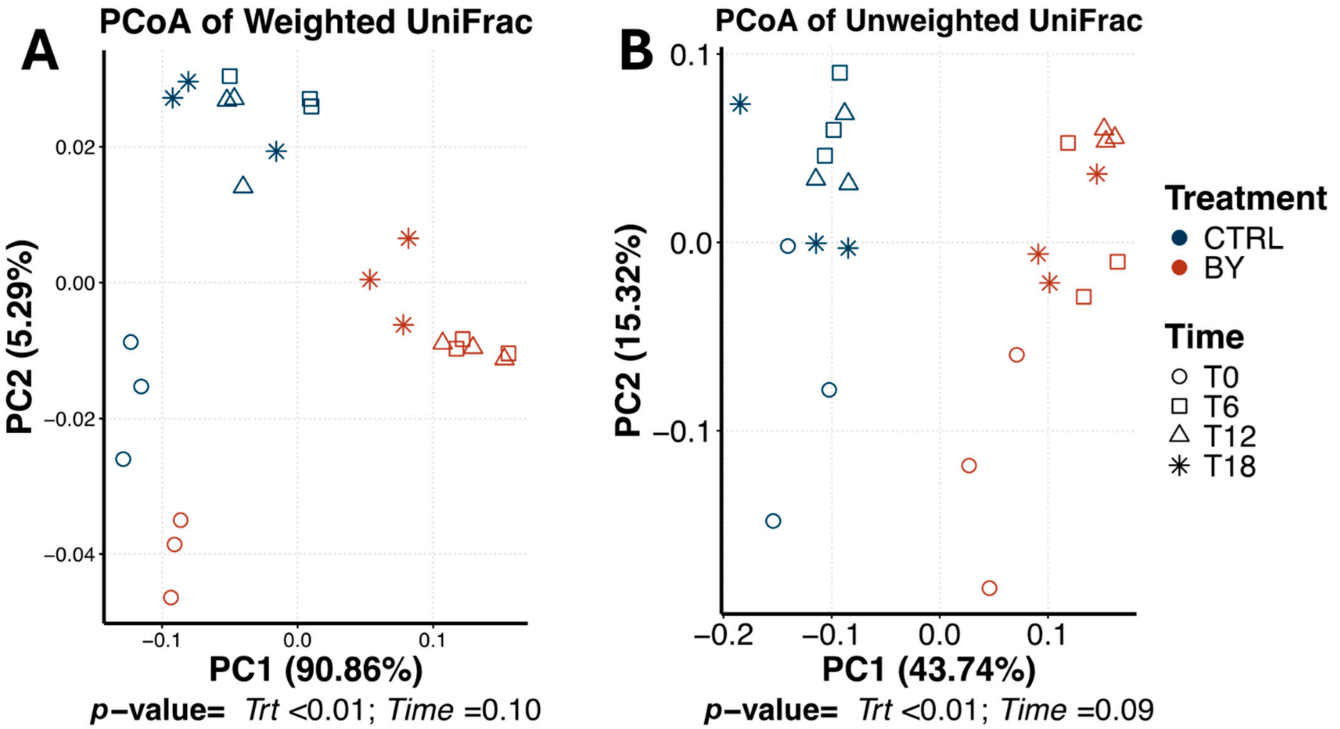

Supplementary Figure 2. Bacterial beta diversity measures of fermentation media during cellulose in vitro fermentation assay using fecal inocula from dogs fed the control diet (CTRL) or diet containing dried brewer's yeast (BY). Principal coordinates analysis (PCoA) plots of weighted (A) and unweighted (B) UniFrac distances revealed two distinct clusters corresponding to each dietary treatment. T0 = immediately after inoculation, T6 = 6 h after inoculation, T12 = 12 h after inoculation, T18 = 18 h after inoculation.

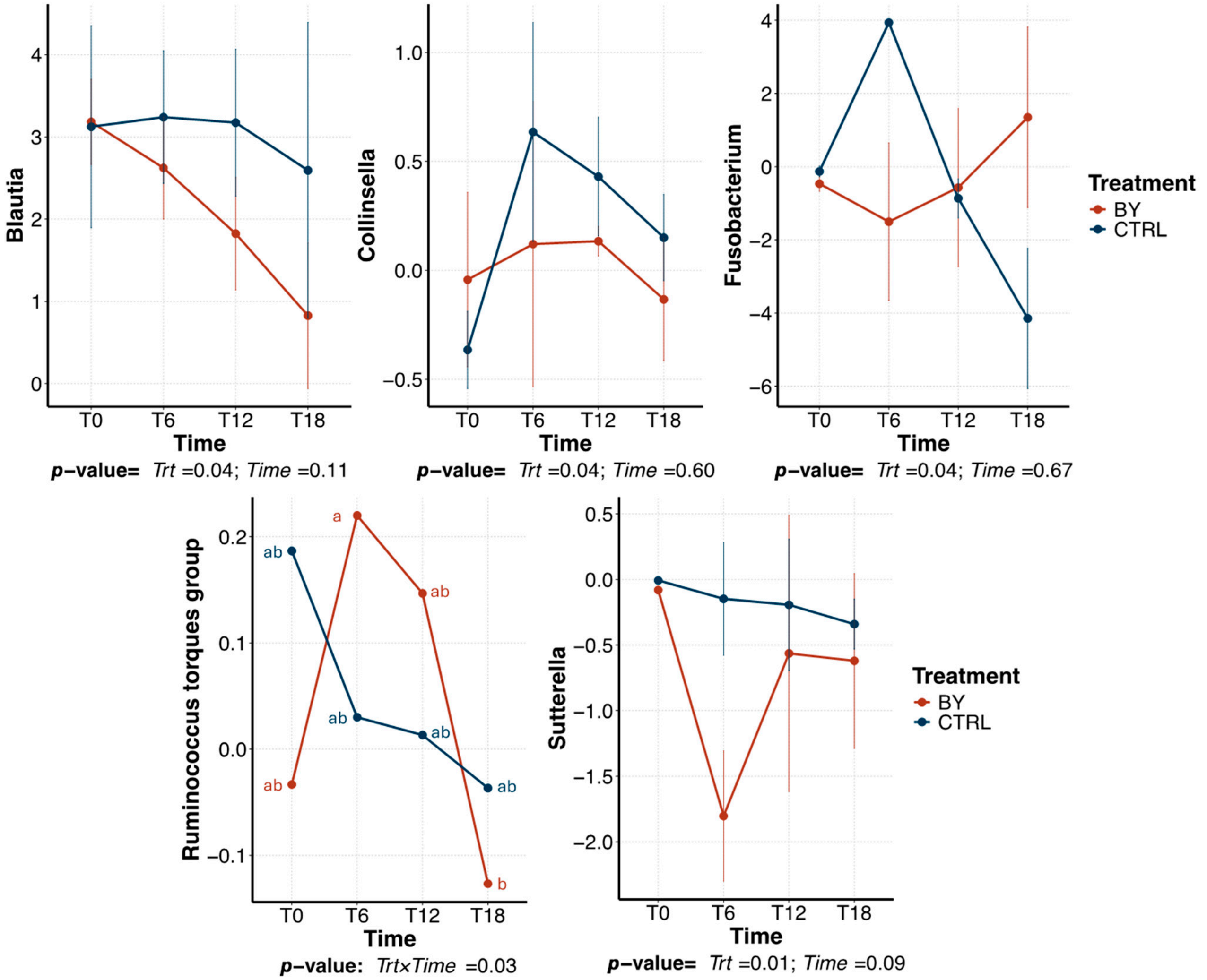

Supplementary Figure 3. Change in relative abundances (% of sequences) of predominant bacterial genera during cellulose fermentation with fecal inoculum from dogs fed the control diet (CTRL) or diet containing dried brewer's yeast (BY). [a-b]Means with different superscript differ ($P \leq 0.05$). T0 = immediately after inoculation, T6 = 6 h after inoculation, T12 = 12 h after inoculation, T18 = 18 h after inoculation.
